# The Role of Ultrasound in the Management of Scrotal Disorders

**Published:** 1992-12

**Authors:** J. McLoughlin

**Affiliations:** Senior Urological Registrar


### The Role of Ultrasound in the Management of Scrotal Disorders

**J. McLoughlin, M.S., F.R.C.S.**
Senior Urological Registrar

### THE NORMAL SCROTUM

The normal testis sonographically appears homogenous in texture, of medium echogenicity and measures between 3-5cm in length and 2 -3cm in antero - posterior diameter. The epididymis appears approximately of the same or slightly greater echogenicity when compared to the testis, albeit a little coarser. Whilst the mediastinum testes can be identified as an echogenic line extending caudally from the upper pole of the testis, neither the tunica albuginea nor various layers of scrotal skin are normally visualised in the absence of a hydrocoele.

### THE PATHOLOGICAL SCROTUM

Conventional high resolution gray-scale ultrasonography is excellent at evaluating scrotal pathology. The particular value of gray-scale ultrasonography is that it may distinguish between intra-testicular and extra-testicular pathology with 90 -99% accuracy (Krone and Carroll, 1985; Narayan et al., 1981). This distinction is of importance as the majority of intra-testicular lesions are malignant whilst extra-testicular lesions tend to be inflammatory, traumatic or benign. Tumours usually appear as discrete hypoechoic or anechoic masses in contrast to inflammatory lesions that appear homogenous and hyperechoic.

The limitation of gray-scale is that it does not assess perfusion, thus restricting its usefulness in cases of suspected torsion. However the recent development of colour Doppler ultrasonography however has overcome this problem allowing both the measurement of blood flow (in colour) in addition to tissue morphology (in gray-scale).

### TUMOUR

The accuracy of ultrasonic distinction between intra- and extra-testicular lesions lies around 90-95% (Krone and Carroll, 1985), the majority of intra-testicular lesions being potentially malignant. Tumours most often appear as hypoechoic lesions however they may assume a diffuse infiltrative appearance involving the entire gland. Among their differential diagnosis includes scrotal abscess formation, infarction, haemorrhage, tuberculous epididymo-orchitis or granuloma formation. It is of special benefit where the underlying testes is impalpable because of a hydrocoele.

The appearance of a testicular tumour depends upon it's histological type. Seminomas account for 40-50% of all germ cell tumours and are almost invariably confined within the tunica albuginea. They appear sonographically as uniformly hypoechoic and only infrequently demonstrate areas of haemorrhage or necrosis. Embryonal cell carcinoma accounts for 20-25% of germ cell tumours and frequently invades the tunica, thereby distorting the testicular contours. It's sonographic appearance is of a poorly circumscribed, hypoechoic mass within the testicle with tunical invasion and contour distortion. In addition, such tumours frequently contain haemorrhagic or cystic areas. Teratomas represent 5-10% of primary testicular tumours and are commonly cystic on ultrasound (containing material such as keratinaceous material, bone or cartilage). Undifferentiated teratomas may also occur, the sonographic appearance reflecting their complex nature with both sonolucent and highly echogenic areas. Approximately 60% of primary testicular tumours are of one histologic type, the remaining 40% demonstrating a mixture of components.

Carcinoma in situ of the testis has been documented in 5% of contralateral testes in patients with germ cell tumours (*Berthelsen et al.*, 1982) and scrotal ultrasound has been shown to be of value as an adjunct to the follow up of the remaining testis in such patients. Ultrasound is also of use in the detection of the occult primary testicular tumour lying within testes that otherwise appear normal on palpation (Bockrath et al., 1983), for example in patients presenting with metastases from an unknown primary source. In a small proportion of cases such lesions may only appear as scar tissue, and as such are thought to represent a "burnt out" primary lesion.

### THE ACUTE SCROTUM

Colour Doppler ultrasound has greatly increased the value of traditional ultrasound in patients presenting with acute testicular pain (*Horstman et al.*, 1991; *Lerner et al.*, 1990)

demonstrating a reduction in blood flow in cases of torsion (*Middletown et al.*, 1989) and the hyperaemia of inflammatory processes (*Horstman et al.*, 1991). *Horstman et al.* (1991) described the characteristic appearance of inflammatory lesions to be hyperaemia associated with an increased number and concentration of blood vessels and a reduction in vascular index. By comparison to gray-scale ultrasound, colour Doppler is both more sensitive and of greater specificity. *Burks et al.* (1990) reporting it to be 86% sensitive and 100% specific in distinguishing torsion from inflammation. Similarly, *Dewine et al.* (1992) studied 20 men with acute scrotal pain with colour Doppler ultrasound. Of 9 who on clinical grounds required surgical exploration, 8 (89%) were correctly predicted by conventional ultrasound while all 11 of those who did not require surgery were correctly identified (100%). These authors proposed that the presence of intra-testicular blood flow correlated well with a successful outcome of conservative therapy.

A limitation of colour Doppler ultrasonography is that testicular neoplasms may mimic inflammation, with hypervascularity being seen with diffusely infiltrating primary testicular tumours or with secondary leukaemic or lymphomatous deposits.

Gray-scale ultrasound may still have a role however, namely as a prognostic indicator in those patients with acute infection but who do not require immediate orchiectomy. *See et al.* (1988) evaluated patients with epididymitis observing that of 21 men with abnormal findings, 1/10 men with epididymal enlargement alone, 2/8 with epididymal enlargement plus hypoechoic testicular lesions (correlating with infection) and all 3 with epididymal enlargement plus non-homogenous testicular appearance (correlating with testicular infarction) required subsequent orchiectomy. In addition, sequential ultrasound may be of benefit in following such patients, those exhibiting progressive changes possibly being candidates for orchidectomy.

### TRAUMA

Injuries to the scrotum can be classified as either penetrating or blunt. Of the two, blunt injuries pose the most problems to the clinician. Ultrasonography is indicated following trauma where scrotal oedema or haematoma preclude palpation of the underlying testes. Haematoma of the scrotal wall layers, the presence of haematocoele, protusion of seminiferous tubules and / or intra-testicular haematoma may all be identified by scrotal ultrasound. Scrotal sonography has also been proposed following trauma in those patients who present with pain but without clinically evident swelling or haematocoele formation as early intervention in the presence of a haematocoele may reduce the orchidectomy rate compared to that of an initial conservative approach and subsequent late exploration (*Martinez - Pereira et al.*, 1991).

### INFERTILITY

The use of scrotal ultrasound in the investigation of infertile males has been described. *Patel* and *Pareek* (1989) studied 200 men, finding 57% having some abnormality including infective signs (19.5%), varicocoele (14%), atrophic testes (13%), undescended testes (1.5%), spermatocoele (1.5%) and hydrocoele (12%). The authors proposed that ultrasonography is of most value distinguishing those patients that may value from surgery (eg. those with a varicocoele) or alternatively who may be candidates merely for careful counselling and consideration for placement in assisted pregnancy programmes (eg. atrophic testes or undescended testes).

### REFERENCES

Berthelsen, J. G., Skakkeback, N. G. and van der Maase, H. *et al.* (1982). Screening for carcinoma in situ of the contralateral testis in patients with germinal testicular cancer. *Brit. Med. J.*: **285**: 1683 - 1686.

Bockrath, J. M., Schaeffer, A. J., Merriel, S. K. *et al.* (1983). Identification of the impalpable testicular tumour. *J. Urol.*: **130**: 355 - 356.

Burks, D. D., Markey, B. J., Burkhard, T. K. *et al.* (1990). Suspected torsion and ischaemia: evaluation with color Doppler sonography. *Radiology*: **175**: 815-821.

Dewine, D. M., Begun, F. P., Lawson, R. K. *et al.* (1992). Colour Doppler ultrasonography in the evaluation of the acute scrotum. *J. Urol.*: **147**: 89-91.

Horstman, W. G., Middletown, W. D. and Melson, G. L. (1991). Scrotal inflammatory disease: Colour Doppler ultrasound findings (1). *Radiology*: **179**: 55-59.

Krone, K. D. and Carroll, B. A. (1985). Scrotal ultrasound. In *Radiology Clinics of North America*, vol 23, 1, Saunders, W. B., Philadelphia. pp. 121-139.

Lerner, R. M., Mevorach, R. A., Hulbert, W. C. *et al.* (1990). Colour Doppler US in the evaluation of acute scrotal disease. *Radiology*: **176**: 355-358.

Martinez - Pereira, Jr., L., Cerezo, E., Cozar, T. M. *et al.* (1991). Value of testicular ultrasound in the evaluation of blunt scrotal trauma without haematocoele. *Brit. J. Urol.*: **69**: 286-290.

Middletown, W. D., Melson, G. L. (1989). Testicular ischaemia: colour Doppler sonographic findings in five patients. *A J R*: **152**: 1237-1239.

Narayan, P., Amplatz, K. and Gondalez, R. (1981). Varicocoele and male infertility. *Fertil. Steril.*: **36**: 92.

Patel, P. J. and Pareek, S. (1989). Scrotal ultrasound in male infertility. *Eur. Urol.*: **16**: 423-425.

See, W. A., Mack, L. A. and Kreiger, J. N. (1988). Scrotal ultrasonography: A predictor of complicated epididymitis requiring orchiectomy. *J. Urol.*: **139**: 55-56.

